# Reassortment of Highly Pathogenic Avian Influenza as a Driver for Zoonotic Spillover, Asia

**DOI:** 10.3201/eid3210.260067

**Published:** 2026-10

**Authors:** Alexander M.P. Byrne, Lorcan Carnegie, Nicola S. Lewis, Ian G. Barr, Erik A. Karlsson, Michelle Wille

**Affiliations:** Worldwide Influenza Centre, The Francis Crick Institute, London, UK (A.M.P. Byrne, N.S. Lewis); Food and Agriculture Organization of the United Nations, Rome, Italy (L. Carnegie); World Health Organization Collaborating Centre for Reference and Research on Influenza, Peter Doherty Institute for Infection and Immunity, University of Melbourne, Melbourne, Victoria, Australia (I.G. Barr, M. Wille); Institut Pasteur du Cambodge, Phnom Penh, Cambodia (E.A. Karlsson); Environment and Infectious Risks Unit, Laboratory for Urgent Response to Biological Threats, Institut Pasteur, Paris, France (E.A. Karlsson)

**Keywords:** influenza, viruses, respiratory infections zoonoses, avian influenza, Asia, H5N1, spillover, reassortment, One Health, clade 2.3.2.1, Asia

## Abstract

Highly pathogenic avian influenza H5Nx viruses remain a major zoonotic threat, yet global attention has focused largely on clade 2.3.4.4b, potentially overlooking major changes within long-endemic H5N1 lineages in Asia. Recent reports from South and Southeast Asia describe the emergence of reassortant clade 2.3.2.1 viruses alongside renewed human infections after apparent prolonged epidemiologic stability. Collectively, those events suggest a regional pattern rather than isolated anomalies. In this article, we argue that reassortment, rather than point mutation alone, might be an underrecognized driver of zoonotic risk in endemic H5N1 lineages and is reshaping those lineages. We examine why such events might be underrecognized in settings with entrenched poultry influenza, identify limitations of current surveillance systems, and call for integrated, real-time approaches linking genomic detection with phenotypic assessment across animal and human health sectors to enable timely risk assessment and coordinated public health action.

Avian influenza viruses (AIVs) remain among the most identifiable but unpredictable threats to both public health and food security (*1*–*4*). Since their emergence in 1996, highly pathogenic avian influenza (HPAI) H5Nx viruses of the A/goose/Guangdong/1/1996 lineage have spread globally, heavily devastating poultry production and repeatedly spilling over into mammals, including humans, often with fatal outcomes. As of August 30, 2025, the World Health Organization (WHO) has reported 990 laboratory-confirmed H5N1 human cases across >25 countries, including 475 deaths (*5*). Since the onset of the HPAI H5Nx panzootic in 2021, the global focus has been on clade 2.3.4.4b, given its dramatic global spread. The focus on clade 2.3.4.4b is warranted given the spillover to and circulation in dairy cattle and the increase in human cases associated with those outbreaks, because those issues raise substantial concerns that the viruses might emerge as a future human pandemic. However, the focus on clade 2.3.4.4b risks obscuring major evolutionary and zoonotic changes within more ancestral but persistent H5N1 clade 2.3.2.1 lineages, which remain enzootic in parts of Asia and receive limited global attention.

## Evolutionary Diversification and Resurgence of Zoonotic Infections in Clade 2.3.2.1 Viruses

HPAI H5N1 clade 2.3.2.1 emerged in 2007 and subsequently spread across Asia and into Europe (*6*,*7*). Continued circulation in Asia led to further classification into subclades 2.3.2.1a, 2.3.2.1b, and 2.3.2.1c in 2014. Because the viruses have continued to evolve, 2.3.2.1c has been further reclassified into subclades 2.3.2.1d–g (*6*,*8*). That diversification reflects regional geography and poultry production systems, such that at present those viruses predominately circulate in live bird markets; clade 2.3.2.1a has predominately been detected in poultry in Bangladesh and India, and 2.3.2.1e circulates in Cambodia, Laos, and Vietnam (*1*,*9*–*11*; A.A. Raut et al., unpub. data, https://www.biorxiv.org/content/10.1101/2025.02.23.638954v1). Beyond 2.3.2.1a and 2.3.2.1e, the only other subclade that continues to be detected (2.3.2.1g) is restricted to poultry in Indonesia (*12**;* T.T. Lam et al., unpub. data, https://www.biorxiv.org/content/10.1101/2025.11.23.690055v1). The remaining 2.3.2.1 subclades were previously found to have circulated predominantly in poultry in Asia; China (2.3.2.1b, 2.3.2.1d, and 2.3.2.1f), Hong Kong (2.3.2.1b and 2.3.2.1c), India (2.3.2.1f), Mongolia (2.3.2.1c), Nepal (2.3.2.1c), Japan (2.3.2.1c), Vietnam (2.3.2.1b), the Middle East (2.3.2.1f), Africa (2.3.2.1f), and Europe (2.3.2.1c), but very few cases have been detected since 2020 (T.T. Lam et al., unpub. data).

Before 2015, HPAI H5N1 virus clade 2.3.2.1 caused 1 human infection across Cambodia, India, and Bangladesh; in subsequent years, the clade became enzootic and ecologically stable in poultry within those countries but was associated with relatively few reported human infections (Figure 1). Since 2023, a measurable rise in human infections caused by clade 2.3.2.1 virus has been observed. During February 2023–November 19, 2025, Cambodia reported 34 HPAI H5N1 cases after nearly a decade without confirmed infections, based on notifications to WHO and sequence data from GISAID (*13*) and GenBank (*14*). In parallel, 6 HPAI H5N1 human cases were reported in India and Bangladesh in 2025 alone (*15*).

**Figure 1:**
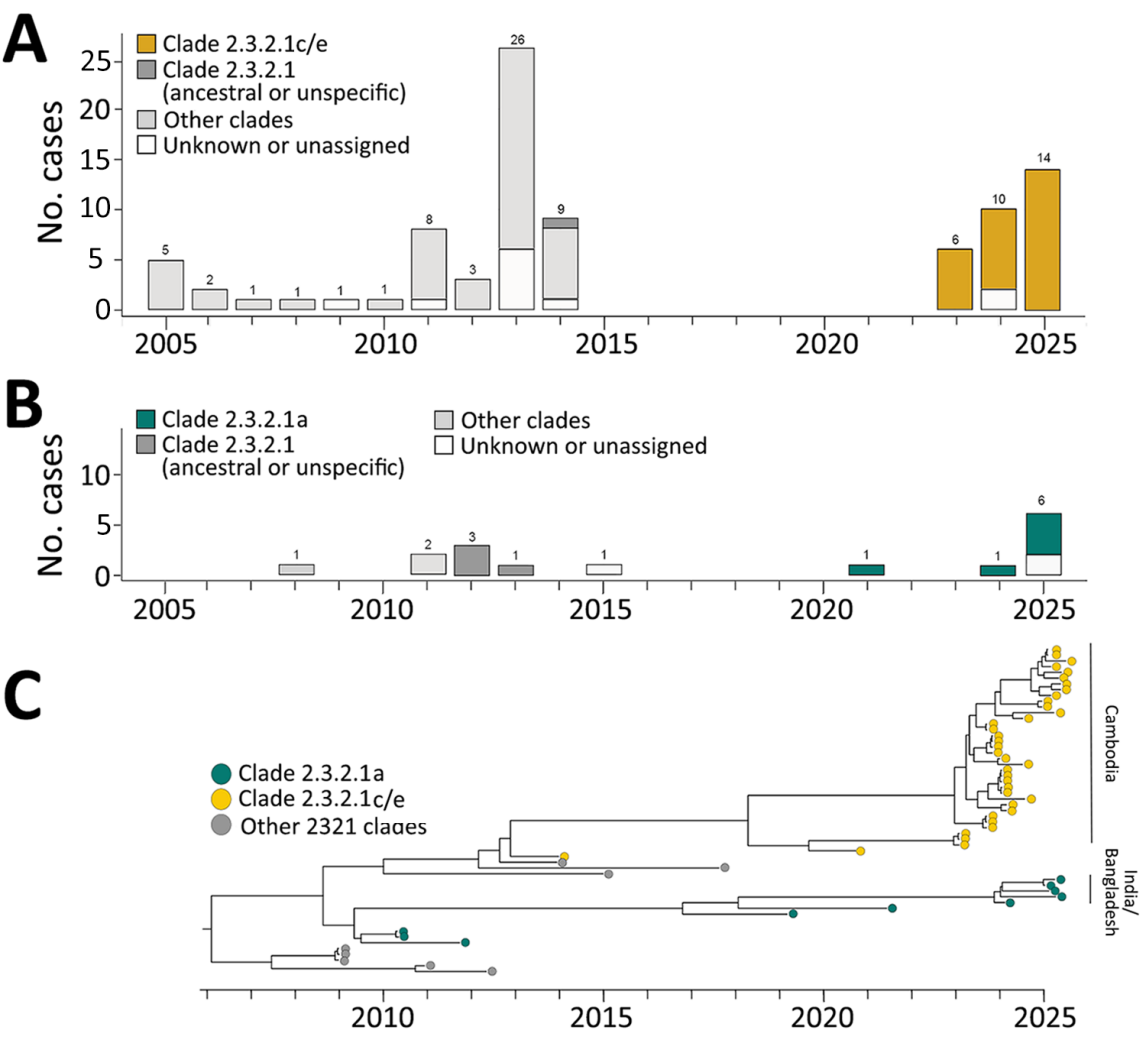
Emergence and human case trends of highly pathogenic avian influenza (HPAI) H5N1 clade 2.3.2.1 viruses in study of reassortment of HPAI as a driver for zoonotic spillover, Asia. A, B) Case notification data of HPAI H5N1 in Cambodia (A) and India and Bangladesh (B), demonstrating the clear resurgence of zoonotic spillover occurring in 2023, driven by reassortant clade 2.3.2.1c/e and 2.3.2.1a viruses. C) Time-scaled phylogeny of the H5 hemagglutinin gene for clade 2.3.2.1 viruses, highlighting the divergence of clade 2.3.2.1a and clade 2.3.2.1c/e.

Alarmingly, all HPAI H5N1 human infections since October 2023 were caused by reassortants from viral backgrounds with distinct ecologic and evolutionary characteristics: long-circulating enzootic clade 2.3.2.1 lineages, HPAI H5N1 clade 2.3.4.4b, and low pathogenicity avian influenza (LPAI). Specifically, in Cambodia, all recent human infections have involved reassortant viruses containing hemagglutinin (HA), neuraminidase (NA), and nucleoprotein (NP) gene segments from clade 2.3.2.1e viruses and internal gene segments likely derived from clade 2.3.4.4b (polymerase basic [PB] 2, matrix protein [MP], nonstructural [NS]) and LPAI (PB1 and polymerase acidic [PA]) viruses (Figure 2) (*11*). This genotype is now dominant in poultry in Cambodia (*11*). In parallel, reassortant clade 2.3.2.1a viruses have caused 2 human cases in India (*15*) and 4 cases in Bangladesh in 2025 (*5*,*15*,*16*) and was detected in a person traveling from India to Australia in 2024 (*9*). Those viruses retained the clade 2.3.2.1a HA, NA, NP, and NS gene segments but had internal gene segments likely derived from a combination of clade 2.3.4.4b (PB2 and MP) or LPAI (PB1 and PA) viruses (Figure 2).

**Figure 2:**
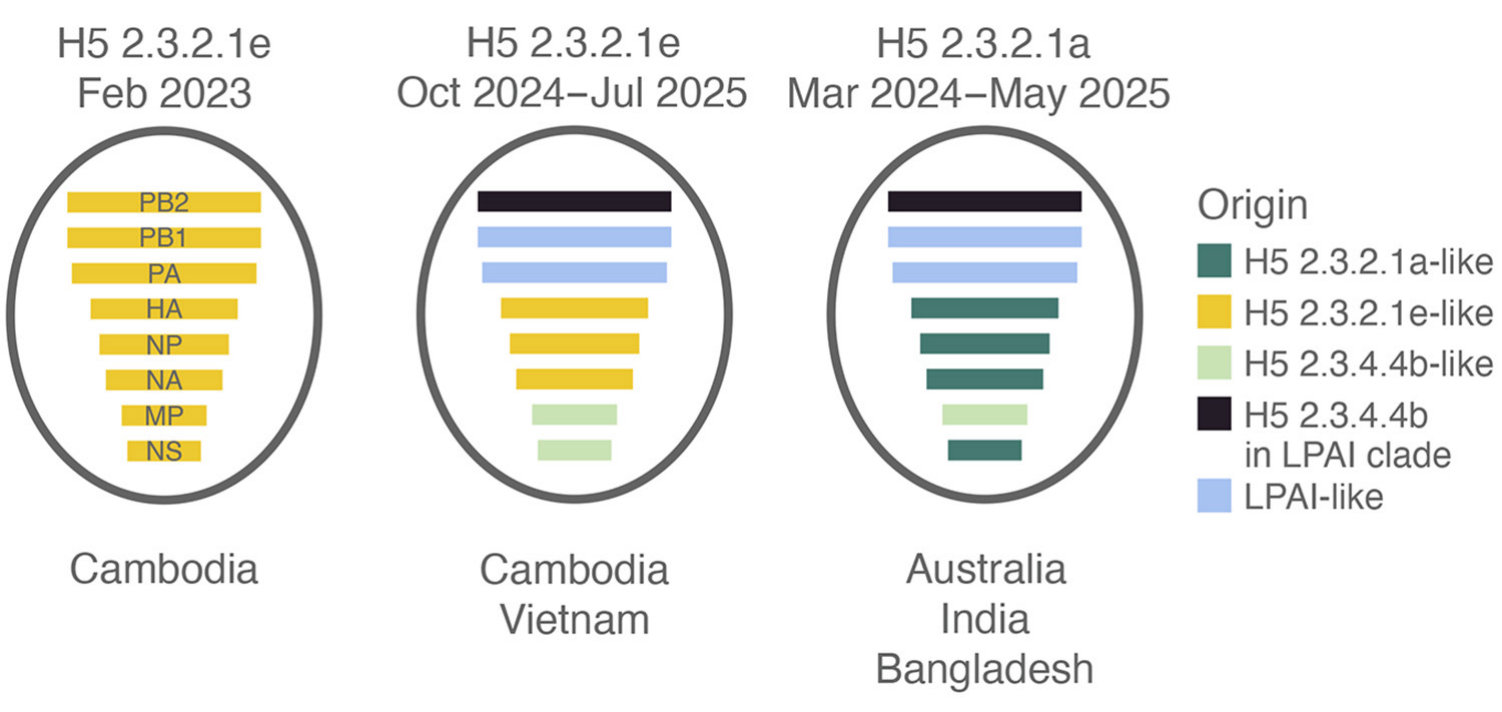
Simplified schematic of the segment constellations of identified human reassortant viruses in study of reassortment of highly pathogenic avian influenza as a driver for zoonotic spillover, Asia. Figure demonstrates the origin of each genomic segment (H5 clade 2.3.2.1a, 2.3.2.1e, 2.3.4.4b, LPAI, or H5 clade 2.3.4.4b within an LPAI clade 2.3.4.4b) by country of detection. Australia detection was in a person traveling to Australia from India. HA, hemagglutinin; LPAI, low pathogenicity avian influenza; MP, matrix protein; NA, neuraminidase; NP, nucleoprotein; NS, nonstructural; PA, polymerase acidic; PB, polymerase basic.

## Possible Increased Zoonotic Risk for Clade 2.3.2.1 Viruses from Underdetected Reassortments

Reassortment is well recognized as the mechanism underlying all human influenza pandemic viruses in the 20th Century (*4*,*17*–*19*). In the context of HPAI H5N1 clade 2.3.2.1, both 2.3.2.1a and 2.3.2.1e clades have undergone reassortment in the past, but available data suggest that, in the decade before the recent reassortants emerged, they were characterized by relatively stable gene constellations (*20*,*21*). In contrast, clade 2.3.4.4b viruses have been marked by frequent reassortment (*22*,*23*; J. Baxter et al., unpub. data, https://www.biorxiv.org/content/10.1101/2025.07.19.665680v2). The emergence of reassortants between clades 2.3.4.4b and 2.3.2.1 viruses might therefore reshape the epidemiology of clade 2.3.2.1 viruses, challenging long-held assumptions about their behavior in poultry systems and their associated zoonotic risk (*11*; W.T. Harvey, unpub. data, https://www.biorxiv.org/content/10.1101/2025.08.20.670882v1).

We hypothesize that the potential epidemiologic changes in HPAI H5N1 clade 2.3.2.1a/e viruses, coinciding with phenotypic changes from the reassortment, might have increased opportunities for zoonotic spillover in Asia. Specifically, we propose that those reassortants emerged and circulated in poultry before they were detected in humans and that they might have gained a fitness or transmission advantage in poultry that increased human exposure. Testing for those potential changes is currently constrained by a key knowledge gap: the gene constellations of viruses circulating in poultry in Asia before the human detections have not been systematically characterized. That gap means neither the origin nor the prevalence of the reassortants can be comprehensively established. Resolving the gap in the short term will require retrospective testing of archived poultry samples collected before the recognized human cases (where available) and, in the longer term, improved surveillance systems. Detecting the reassortant constellations in such samples would support previous circulation in poultry; its absence would instead point to a fitness advantage for mammalian adaptation rather than poultry transmission. To our knowledge, no such systematic retrospective screening has yet been reported, and it should be prioritized to clarify the directionality of spillover.

Key research in epidemiology and phenotypic characterization is needed to test our hypotheses. So far, we have not observed a rise in poultry prevalence before human infections caused by the reassortants; however, in the HPAI H5N1 enzootic poultry systems where outbreaks are often underreported, confirming whether the true prevalence has increased is challenging. To understand that question, more timely and sensitive surveillance and phylogenetic and epidemiologic analyses are needed. We also do not know whether reassortment itself caused a phenotypic advantage in causing zoonotic infections. Further phenotypic characterization is needed to elucidate the potential mechanisms through which these reassortants gained the ability to cause zoonotic infections.

Although a direct causal link between those specific reassortment events and the increase in recent human infections in Asia has not yet been demonstrated, current research in Cambodia and available sequence data from other countries, such as Bangladesh, demonstrate a clear association (*9*,*11*). It is of particular concern that those reassortants combine gene segments from viral backgrounds with distinct ecologic and evolutionary characteristics. Therefore, we argue that, in general, reassortment is a key evolutionary mechanism shaping zoonotic risk for HPAI H5N1. Accordingly, we highlight the gap in current surveillance systems and propose an improved system to detect and interpret avian influenza virus strains of zoonotic risk.

## Limited Real-Time, Integrated Approach to Detecting and Interpreting Risk

Current surveillance systems have key limitations in detecting and interpreting reassortment events. The issue is not only whether a reassortant virus is detected but whether its significance is recognized early enough to enable appropriate mitigation strategies to be implemented. In many endemic poultry settings, surveillance still relies heavily on clade-level reporting and targeted monitoring of known mammalian-adaptive mutations or antigenic markers (*24*–*27*), rather than routine whole-genome sequencing (WGS) and integrated genotype-level analysis. That factor can obscure substantial underlying genetic change, creating a false impression of continuity even when viruses have acquired novel internal gene constellations with considerable phenotypic consequences. Reassortants might therefore be underdetected or detected only after they are associated with human cases. Even when WGS data are available, genetic constellations alone rarely reveal when reassortment occurred, how long the reassortant has circulated, or whether it has altered viral fitness, host range, transmissibility, or antigenicity. Those questions require timely phylogenetic analysis, phenotypic characterization, antigenic testing, and epidemiologic investigation. Strengthened surveillance capacity after the COVID-19 pandemic might partly explain increased detection of human infections but does not remove the need for systems capable of identifying sustained influenza reassortment events earlier and assessing whether they represent a meaningful change in zoonotic risk.

## Improved Surveillance for Detecting, Analyzing, and Interpreting Reassortment

A reimagined approach to monitoring zoonotic risk requires real-time tracking of major and sustained reassortment events, supported by systems that can detect, share, and interpret genomic, phenotypic, and epidemiologic data as those events unfold. Near–real-time WGS and data sharing are essential for identifying emerging reassortant viruses, particularly at high-risk interfaces where wild and domestic birds interact, including live bird markets, backyard poultry systems, farms, and wetlands. Those steps would improve on current systems by moving beyond clade-level reporting and targeted screening for known mammalian-adaptive markers, which can miss key changes in internal gene constellations. Newly emerging genotypes should be assessed through phylogenetic analysis, epidemiologic investigation, and routine phenotypic characterization. In addition, emerging computational approaches, such as CoalRe (*28*) and TreeSort (*29*), could help reconstruct the evolutionary history of reassortment events, estimate their timing and persistence, and guide surveillance and control strategies to assist with detecting emerging novel genotypes. However, the outputs of such tools must be evaluated in the context of epidemiologic and phenotypic data.

Once sustained reassortment is identified, phenotypic testing is needed to evaluate the biological risk posed by the viruses, assess whether they remain matched to existing candidate vaccine viruses, and contextualize the genomic data. Ideally, that step should occur even in the absence of recognized human cases, enabling timely risk interpretation before zoonotic infections are detected. This factor is particularly key because available evidence suggests that reassortant viruses might already have been circulating in poultry in Bangladesh and Cambodia (*11*) before the first recognized reassortant-associated human cases. Testing whether reassortment increases zoonotic risk would require comparisons with other circulating poultry genotypes, estimates of how frequently similar reassortants arise, mammalian infection data, phenotypic characterization, and analyses of when reassortment occurred and how long reassortant viruses circulated before detection. Those tactics could help determine whether reassortment has altered viral fitness, host range, transmissibility, antigenicity, or zoonotic risk.

To summarize, surveillance for reassortants with potential zoonotic risk should be structured as a linked and sometimes iterative pathway: sample collection; laboratory testing, including PCR, WGS, data sharing, virus isolation, and phenotypic characterization; data collation and analysis to enable risk interpretation; and wider data sharing to the scientific community and public detailing the perceived risk and appropriate mitigation measures. Systematic sampling across high-risk interfaces would improve the chance of detecting reassortants before they are recognized through human infections. PCR and WGS would enable novel gene constellations to be identified and compared with other circulating poultry genotypes, whereas virus isolation and phenotypic characterization would help determine whether those viruses show altered replication, host range, antigenicity, vaccine–virus match, or other traits relevant to zoonotic risk. Data collation and WGS phylogenetic/phylodynamic analyses that have been designed to track reassortment patterns should then be used to estimate how frequently similar reassortants arise, when reassortment occurred, how long reassortant viruses have circulated, and whether they are associated with mammalian infections or changes in poultry virus dynamics. For that process to work, surveillance systems need sustained sampling, sequencing capacity, virus isolation facilities, standardized phenotypic assays, analytical tools, trained personnel, interoperable databases, and timely data-sharing mechanisms. In that framework, the hypothesis that reassortment has increased the zoonotic capacity of circulating viruses can be tested directly, rather than suggested only after an increase in human infections is detected.

Ultimately, achieving this vision requires integrated cross-sector systems rather than isolated or duplicated efforts. To enable timely phenotypic analyses, laboratory access to viruses is essential and should be supported by routine peacetime virus-sharing frameworks and predefined decision points for triggering further investigation that can feed into existing initiatives such as the WHO Tool for Influenza Pandemic Risk Assessment (*30*). Those activities, from field surveillance to phenotypic characterization, must also be conducted in a coordinated manner across multiple stakeholders to prevent unnecessary duplication, particularly given the limited resources available, while also ensuring rapid identification and assessment of novel and emerging viruses. Effective mechanisms for joint risk assessment and 2-way genomic data exchange between animal and public health authorities are essential to ensure that emerging genetic findings trigger proportionate and coordinated One Health responses, including clarity on whether the genotypes or amino acid changes detected in human outbreaks reflect those circulating in animal populations. Ideally, surveillance systems would be integrated across animal, human and environmental sectors through interoperable platforms with standardized metadata, enabling rapid identification of emerging genomic constellations and direct comparison with zoonotic cases. Without systems that connect animal and human data streams in real time, reassortant or adaptive events will continue to be detected retrospectively or with substantial delay, increasing the risk for onward human transmission.

In conclusion, the perceived resurgence of HPAI H5N1 infections in humans caused by reassortant clade 2.3.2.1 viruses in South and Southeast Asia is more than an epidemiologic footnote; it is a warning. Such events illustrate how reassortment within long-overlooked Asia HPAI H5N1 lineages is reshaping zoonotic risk, potentially driven by intensifying poultry production, shifting wild bird migration patterns, and intensively shared human–animal interfaces. Yet current surveillance systems are not configured to detect such evolutionary shifts in real time. Unless surveillance is reimagined to capture and interpret reassortment as it occurs through integrated One Health approaches that connect animal, environmental, and human data streams and translate genomic signals into coordinated action, the next warning might not appear in a dataset or sequence repository, but instead as an outbreak already beyond our control.

Appendix Additional information about reassortment of high pathogenicity avian influenza as a driver for zoonotic spillover, Asia.
