# Supplementary material for: Reassortment of Highly Pathogenic Avian Influenza as a Driver for Zoonotic Spillover, Asia
Source: Emerg Infect Dis. 2026 Oct;32(10):1537–42. doi: 10.3201/eid3210.260067 (PMC13632839; doi:10.3201/eid3210.260067)
Supplement: Supplementary file 1 [file 26-0067-Techapp-s1.pdf]

Article DOI: https://doi.org/10.3201/eid3210.260067

EID cannot ensure accessibility for supplementary materials supplied by authors. Readers who have difficulty accessing supplementary content should contact the authors for assistance.

Reassortment of Highly Pathogenic Avian Influenza as a Driver for Zoonotic Spillover, Asia

Appendix

To determine the origins of the 2.3.2.1 human cases, we combined those with a dataset of influenza A genomes with collection dates from January 1, 1996, to May 20, 2025. We refined that dataset to remove all sequences with <90% segment coverage using SeqKit version 2.7.0 (*1*) and then clustered it using CD-HIT-EST version 4.8.1 (*2*) with a 95% identity threshold. We then aligned sequences and used them to generate time-resolved maximum-likelihood phylogenies on a per segment basis using the Augur bioinformatic toolkit version 24.1.0 (*3*) and visualized them using Auspice (*3*). We downloaded all HA sequences of clade 2.3.2.1 from humans in GISAID (*4*) (Figure 1, panel C). We aligned sequences using MAFFT (*5*) within Geneious Prime (https://www.geneious.com). We performed a linear regression of root-to-tip distances against year of sampling using TempEst (*6*) and constructed a time-scaled phylogeny using BEAST version 1.10.4 (*7*,*8*).

All influenza genome sequences and associated metadata supporting this study are publicly available through the GISAID EpiFlu database. The datasets can be retrieved using the GISAID EPI_SET identifiers or their corresponding persistent digital object identifiers (DOIs). Each EPI_SET provides access to the underlying EPI_ISL accession numbers and complete contributor information, including virus name, collection date, country or territory of origin, originating and submitting laboratories, and contributing authors. Users of these data should acknowledge all originating and submitting laboratories and contributors in accordance with GISAID's terms of use.

GISAID dataset identifier	Persistent DOI	Number of viruses	Collection-date range	Countries and territories represented
EPI_SET_260625oa	https://doi.org/10.55876/gis8.260625oa	37	8 September 2017–25 July 2025	7
EPI_SET_250909as	https://doi.org/10.55876/gis8.250909as	8,893	1 January 1996–4 August 2025	105

References

1. Shen W, Le S, Li Y, Hu F. SeqKit: A Cross-Platform and Ultrafast Toolkit for FASTA/Q File Manipulation. PLoS One. 2016;11:e0163962. PubMed https://doi.org/10.1371/journal.pone.0163962

2. Fu L, Niu B, Zhu Z, Wu S, Li W. CD-HIT: accelerated for clustering the next-generation sequencing data. Bioinformatics. 2012;28:3150–2. PubMed https://doi.org/10.1093/bioinformatics/bts565

3. Hadfield J, Megill C, Bell SM, Huddleston J, Potter B, Callender C, et al. Nextstrain: real-time tracking of pathogen evolution. Bioinformatics. 2018;34:4121–3. PubMed https://doi.org/10.1093/bioinformatics/bty407

4. Shu Y, McCauley J. GISAID: Global initiative on sharing all influenza data - from vision to reality. Euro Surveill. 2017;22:22. PubMed https://doi.org/10.2807/1560-7917.ES.2017.22.13.30494

5. Katoh K, Misawa K, Kuma K, Miyata T. MAFFT: a novel method for rapid multiple sequence alignment based on fast Fourier transform. Nucleic Acids Res. 2002;30:3059–66. PubMed https://doi.org/10.1093/nar/gkf436

6. Rambaut A, Lam TT, Max Carvalho L, Pybus OG. Exploring the temporal structure of heterochronous sequences using TempEst (formerly Path-O-Gen). Virus Evol. 2016;2:vew007–007. PubMed https://doi.org/10.1093/ve/vew007

7. Drummond AJ, Rambaut A. BEAST: Bayesian evolutionary analysis by sampling trees. BMC Evol Biol. 2007;7:214. PubMed https://doi.org/10.1186/1471-2148-7-214

8. Suchard MA, Lemey P, Baele G, Ayres DL, Drummond AJ, Rambaut A. Bayesian phylogenetic and phylodynamic data integration using BEAST 1.10. Virus Evol. 2018;4:vey016. PubMed https://doi.org/10.1093/ve/vey016
